# The views of New Zealand general practitioners and patients on a proposed risk assessment and communication tool: a qualitative study using Normalisation Process Theory

**DOI:** 10.1186/s43058-021-00120-1

**Published:** 2021-02-10

**Authors:** Sharon Leitch, Alesha Smith, Sue Crengle, Tim Stokes

**Affiliations:** 1 University of Otago Medical School, Dunedin, New Zealand; 2 School of Pharmacy, University of Otago, Dunedin, New Zealand

**Keywords:** Normalisation Process Theory, Communication, Risk, Medication harm, General practice, Equity

## Abstract

**Background:**

Communicating risks of medication harm and obtaining informed consent is difficult due to structural barriers, language and cultural practices, bias and a lack of resources appropriately tailored for the health literacy of most patients. A decision support tool was proposed to alert prescribers of risk and provide tailored information for patients to facilitate informed decision-making with patients and their whānau (family) around medication use. Patient and prescriber co-design was used to ensure the tool was designed to best meet the needs of end-users and avoid increasing health inequity. This paper describes the first stage of the co-design process.

**Method:**

Normalisation Process Theory (NPT) was used to prospectively evaluate the tool. Semi-structured interviews were held with fifteen patients (five Māori, five Pasifika and five NZ European) and nine general practitioners (two Māori and seven European).

**Results:**

Three themes were identified, which related to the three NPT concepts most relevant to developing the tool. Theme 1 (coherence: meaning and sense making by participants) explored participants’ understanding of prescribing safety, medication harm and risk, which is based on experience. Patients want as much information as possible about their medications and risk, but doctors find it difficult to communicate that information. Theme 2 related to the NPT concept of cognitive participation (commitment and engagement by participants) explored what participants thought about a prescribing decision support tool. Participants were cautiously optimistic, but worried about potential harm arising from its use. They also identified requirements for the tool and features to avoid. Theme 3 describes the collective action required for successful implementation of the tool; namely, culturally safe and trustworthy doctor-patient relationships.

**Conclusion:**

Patients and general practitioners provided different perspectives when prospectively evaluating the proposed risk assessment and communication tool. This co-design research identified important pre-requisites for the tool and features to avoid and novel ideas for the proposed tool. Overall participants supported the development of the proposed risk assessment and communication tool, but identified the important role that doctor-patient relationships would play to ensure successful implementation. The use of Māori and Pacific languages in the proposed tool may enhance engagement and understanding.

**Supplementary Information:**

The online version contains supplementary material available at 10.1186/s43058-021-00120-1.

Contributions to the literature

- A novel electronic decision support tool for use in New Zealand general practice aims to assess patient risk of medication harm and improve risk communication and shared decision-making.
- Patient and general practitioner co-design aims to anticipate implementation issues, improve the tool’s utility and mitigate health inequities arising from its use.
- This paper describes the use of Normalisation Process Theory as a way to aid the prospective evaluation of the proposed tool by general practitioners and patients.
- Normalisation Process Theory enabled exploration of the concepts of safe prescribing, medical autonomy and cultural safety in the context of New Zealand general practice.

## Background

Prescribing medication presents a tension between risks and benefits. Prescribers have a legal and moral obligation to ensure patients are fully informed about those risks and benefits [1–4]. Obtaining truly informed consent can be challenging due to structural barriers, language, differing cultural practices and expectations, bias and a lack of resources appropriately tailored for the health literacy of most patients [5–7].

Health inequities are potentially avoidable differences in health between peoples of different social groups [8]. The founding document of Aotearoa New Zealand (NZ), is Te Tiriti O Waitangi (The Treaty of Waitangi), which, amongst other things, enshrines the concepts of equity and protection of Māori (the Indigenous people) [9–11]. Health inequity in NZ arises from the corrosive effects of colonisation and racism [5, 12–15]. Aspirational goals to “improve, promote and protect the health and wellbeing of New Zealanders [16]” have done little to address the inequity experienced by Māori and Pasifika (people living in NZ who identify as Pacific peoples, including Samoan, Cook Islands Māori, Tongan, etc.) [17].

Māori, Pasifika and people who experience socioeconomic deprivation bear a greater burden of disease and have worse health outcomes across a broad range of health conditions in NZ, including birth outcomes, rheumatic fever, meningococcal disease, long term conditions, multimorbidity and cancer [18–24]. These populations experience disproportionately high adverse event rates, including premature mortality, injury, disability and harms arising from healthcare [5, 17, 25–28]. They paradoxically experience both under-prescribing of appropriate medications, higher prescribing of inappropriate medications [29] and higher rates of polypharmacy [30, 31].

Primary health services provide the majority of healthcare in NZ [11], typically requiring out-of-pocket co-payments, as do prescription medications [32]. The 2018–2019 NZ Health Survey found that similar proportions of Māori, Pasifika and NZ European had attended a general practitioner (GP) in the previous 12 months and that the mean number of visits was higher in Māori (age sex standardised rate ratio 1.22) [33]. However, a higher proportion of the Māori and Pasifika populations reported unmet need for primary health care due to the cost of primary health care over the same period (21.9% of Māori and 19.4% of Pasifika vs. 12.7% of European/Other) [33]. A higher proportion of Māori and Pasifika (11.8% of Māori and 14.0% of Pasifika vs. 4.2% of European/Other) reported not having a prescription filled because of the cost [33]. Overrepresentation of Māori and Pasifika in lower socio-economic groups compounds inequity [12, 34].

Health literacy is the ability to obtain, process and understand health information in order to make informed and appropriate health-related decisions [35]. Low levels of health literacy are associated with worse healthcare outcomes [36]. New Zealanders typically have low levels of health literacy—over half of adults surveyed had skills “insufficient to cope with the health literacy demands they typically face” [7]. The proportion of the Māori population with low health literacy levels is higher with 80% of Māori men and about 75% of Māori women experiencing low levels of health literacy [7]. While policy and public programmes may address health literacy at the national level, clinicians are responsible for communicating health information so patients can understand, whatever their health literacy level [37].

Decision support tools can improve patient knowledge of their options and expectations of outcomes, support patient participation in shared decision making and improve communication between patients and clinicians [38]. Decision support tools available in NZ primary care either focus solely on medication interactions or algorithms for specific medication use. A novel tool which integrates these concepts would potentially address some of the above issues. The development of such a tool was proposed. The tool would alert prescribers to medication risk based on potential interactions and patient factors (e.g. renal function), and provide both clinician decision support and patient information, thus facilitating communication and supporting informed decision-making. The aim of this study is to determine what potential users of the tool (patients and GPs) think about the proposed tool. It is hoped this process will help identify unforeseen issues, such design features that could exacerbate health inequities or be culturally unsafe [39].

### Theoretical framework

This research utilises an implementation science approach. Implementation science has the capacity to increase the impact of health disparity research and mitigate inequities due to its broad focus on all aspects of implementation, from health policy to bedside [40–42]. Further, implementation science theories can provide a framework for the collection and analysis of data and help explain the findings [43]. We have chosen to use one particular implementation science theory: Normalisation Process Theory (NPT). NPT bridges the translational gap between research evidence and practical implementation and is comprehensive, flexible and has a strong focus on participatory co-design [43–46]. NPT has been used for research involving ethnic minority populations and to explore issues of equity [47–50]. NPT provides a useful framework for researchers to anticipate implementation issues while designing a complex intervention and its evaluation [44–46, 51]. Early use of NPT was initially in eHealth interventions; however, its use has spread well beyond this field [44–46]. NPT considers implementation as a social process which requires ongoing work by the parties involved and is divided into four domains, outlined in Table 1: Coherence, Cognitive Participation, Collective Action and Reflexive Monitoring. Minimising the amount of work required to use the tool and any potential disruption to workflow will help ensure that the tool is actually used [53].

**Table 1:** Normalisation process theory (NPT) concepts in developing an e-tool

NPT Concept [52]	Example interview questions^**a**^
**Coherence** Is the intervention meaningful for participants? Establish shared definitions and understanding of both the problem and the potential intervention	**Patient questions:** What does harm from medicine mean to you? What does risk from medicine harm mean? When do you think it’s important to know about your risk from medication? Do you think it’s important to discuss medication risk with your GP? **GP questions:** What prompts you to consider assessing a patient’s risk from their medication? How confident do you feel explaining medication risk to patients?
**Cognitive Participation** Do participants think the intervention is a good idea? Establish whether patients and doctors are committed to engage with this tool	**Patient questions:** Do you think the proposed MedKōrero tool, to assess risk and improve communication about that risk, will help you/your whanau make decisions about treatment? What kind of impact would a tool like this have for you/your whanau when you are deciding on a treatment option? **GP questions:** Do you think the proposed MedKōrero tool, to assess risk and communicate that risk to patients, will promote shared decision making? Would it be helpful to your day-to-day work?
**Collective Action** What work needs to be done to implement this new intervention? Ascertain the likely work participants will need to do to in relation to the tool, in order to learn what features the tool requires in order to minimise additional work.	**Patient questions:** What would promote its use? What would be a barrier to its use? **GP questions:** What kind of impact would a tool like this have in your clinical setting? What would promote its use? What would be a barrier to its use?
**Reflexive Monitoring** Ascertain the likely impact of the tool, in order to develop the tool to enhance positive impact and minimise negative impact.	**Patients and GPs:** Can you think of potential system-wide effects of using this tool? What would be the intended and unintended consequences

^a^Appendix 2 contains the complete interview guide

## Method

The consolidated criteria for reporting qualitative research guidelines (COREQ) were used to prepare this article (see Appendix 1 for full methodological details) [54].

Stakeholder co-design was planned to ensure the tool was designed to best meet the needs of end-users [55]. Semi-structured interviews were conducted using a topic guide (Appendix 2), which was informed by the domains of NPT most relevant to prospectively evaluating a tool: Coherence, Cognitive Participation and Collective Action. Participants were essentially co-opted to participate in the cognitive work of developing ideas around the tool during the course of the interview. Participants were advised of the broad overview of the tool in the advertising material, the participant information sheet, the consent form and verbally at the start of the interview as follows. “We want to develop and trial a tool, to alert primary care prescribers when patients are at increased risk of harm from medication. We hope this tool will facilitate communication about medication risks and empower shared decision-making about medication use between patients and prescribers. We want to talk to patients and prescribers about their opinions about a tool like this, to help develop a tool that is going to best help both patients and prescribers.” No prototype was presented as it was consider that may overly influence participants’ comments.

The prescriber (GP) interview framework was pilot tested with a GP prescriber by SL and TS observing, who then provided feedback on further iterations of the topic guide. No changes were made as a result of the pilot interview and it was not included in the analysis. The topic guides were used flexibly to allow participants to construct their accounts in their own terms.

### Recruitment

A purposive sampling approach was taken for recruitment of both doctors and patients, with the aim of recruiting ethnically diverse samples, particularly of patients. Participants were recruited by personal contact and Facebook group pages. The study team anticipated that we would not need any more than 15 patient or GP interviews to reach data saturation in each group. See Appendix 1 for further details of the recruitment strategy.

SL was identified as a GP and a PhD candidate and recruited all participants. Participants received information about the study and signed a consent form prior to their interview.

### Data collection

SL interviewed all participants once, either in person, by phone or videoconference between 8 April and 2 July, 2019. Face-to-face interviews were conducted in a place of the participants choosing; either in a University office, a café, the patient’s workplace or home. All interviews were conducted in English. Interviews were recorded and transcribed. Interview field notes were taken during phone and videoconference interviews only. One prescriber phone interview could not be recorded; this interview was written up from detailed interview notes. Two prescriber participants supplemented their interview by emailing further information or background documents after the interview.

Nine doctor and 15 patient interviews were undertaken. Data was transcribed and preliminary analysis occurred concurrently with the interviewing process. Data saturation was reached before the conclusion of these interviews with no new ideas being discussed by participants.

### Data analysis

A deductive thematic analysis was conducted using the framework method [56]. Interviews were coded by SL, assisted by NVivo 11 software, into the three relevant NPT domains. Interpretation of the data was an iterative process which was led by SL, with review of the codes, subcategories, categories and themes by TS.

## Results

Fifteen patients (five Māori, five Pasifika and five European patients) and nine doctors (two Māori and seven European general practitioners) were interviewed (Table 2).

**Table 2:** Demographic details of study participants

	Patients	Doctors
**Gender**		
Male	4	6
Female	11	3
**Age**		
< 50	8	4
≥ 50	7	5
**Ethnicity**		
European	5	7
Māori	5	2
Pasifika	5	0
**Location**		
Rural	0	2
Urban	15	7

**Figure 1:**
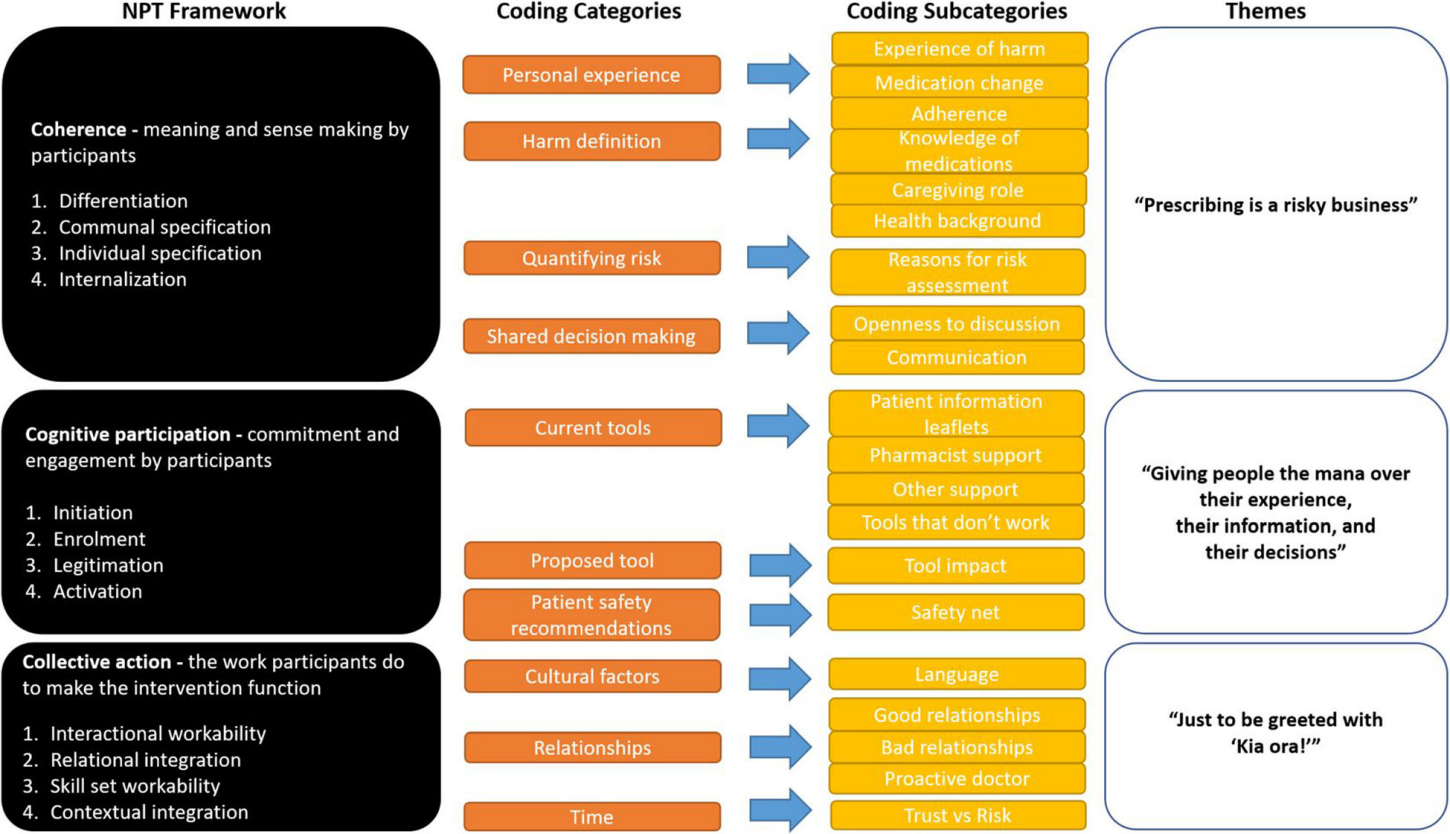
Fig. 1 Relationship between NPT framework, coding categories and themes

Figure 1 outlines the Normalisation Process Theory (NPT) framework together with the categories and subcategories developed from the interview data and how the coding frame relates to the study themes. Illustrative participant quotes are presented.

### Theme 1—“Prescribing is a risky business” [Coherence]

Recognising the tension between the risks and benefits of medication was critical for understanding the rationale for the project, and both doctors and patients expressed cognisance of this, drawing on their experience of medication harm. Doctors based their understanding of risks and benefits on their clinical experience:

“Medications are always a balance of gain or whatever you’re trying to treat balanced against risks of potential side effects.” Doctor 9 (European female)

Patients, on the other hand, referenced their personal or whānau (family) experiences:

“With the preventer I take, you do have a chance of getting oral thrush in your mouth. So that has happened to me a couple of times. Yeah. And it's a pain in the butt, but it’s better than having an asthma attack so I knew that it might happen. And when it did, I was like, well, this is the trade-off. I prefer not to have an asthma attack.” Patient 1 (Māori female)

“I think the more medication you take the more risk of things interacting. I mean, some of the things I take, it says if your kidney function’s going down don’t give it, and I think, I’m already on it. They know I’m on it. And, you know, I accept that there are just risks you’ve got to take.” Patient 6 (European female)

The doctors reported tailoring the amount and type of information to share with their patients depending on their perception as to what the patient would like to know. Doctors relied on their knowledge of their patients to determine what level of shared decision-making was attempted:

“I make a rough assessment depending on how well I know them, I suppose, about how much they understand. It’s a dynamic thing with all those things as I go through thinking about it and discussing what we do, depending on how they respond, I can go a little bit differently. It’s not a static thing.” Doctor 7 (European male)

Doctors found communicating information about medication and medication risks difficult. Barriers to communication reported included time, the challenge of presenting information at an appropriate health literacy level and a perception that some patients are not interested in this information. Doctors typically focussed on communicating the important risks:

“It takes a long time to explain small risks of harms so I tend to filter out the important ones, or the ones I feel are important.” Doctor 3 (European male)

“Our perception of risk is always very different to theirs. And also trying to communicate percentages, or you know, numbers needed to treat, all those sorts of concepts are very difficult for people to take on board.” Doctor 9 (European female)

“Health literacy is a real skill I think in connecting with people and communicating on their level... you don’t always get that right. So it’s as much for the aggressive, in a rush person who is frustrated at waiting thirty minutes and just wants to pick their pills up and get out of there, as much as it is for the person who can’t read and write, and has difficulty in conceptualising how medications work, and what we mean by risk.” Doctor 5 (European male)

In contrast, patient participants were keen to have as much knowledge about their medications as possible. Patients frequently wanted more information than their doctor or pharmacist provided. They were happy to find their own information, but were concerned about the quality of the information they found on the internet:

“I have admit that after five years I’ve only got a vague idea what my drugs actually do... I want to be more informed as to what my medication is…. I would like to be more empowered. To know what it is I’m taking and why I’m taking it.” Patient 2 (Māori male)

“You can research a lot, but authenticating what’s genuine and what isn’t as well can also be huge.” Patient 9 (Māori female)

### Theme 2—“Giving people the mana over their experience, their information and their decisions” [Cognitive Participation]

The idea of the proposed tool was met with qualified enthusiasm from both patients and clinicians. Patients and clinicians wanted to include various elements as part of a risk assessment and communication tool (Table 3). Participants, both doctors and patients, were cautiously optimistic that the proposed tool would be beneficial. Doctors felt the tool had potential to reduce their workload, as having tailored risk information readily accessible could save time and be a useful resource for both patients and clinicians. Conversely, they were frank to admit they would reject anything perceived to impact negatively on their workflow or on the doctor-patient relationship. Doctors thought that if the tool worked well, it could actually prevent medication related harm, reducing patient morbidity and pressure on the health system:

“I guess you’d hope the hospital presentations would be lessened because of it, given the number of hospital admissions that are related to medication harm.” Doctor 3 (European female)

“Realistically the consequences could be enormous if harm was prevented, and hospital admissions were saved.” Doctor 6 (European male)

**Table 3:** Summary of what participants want in a prescribing and communication tool

**Doctors and patients want**	
• A trustworthy, endorsed tool • A user-friendly tool that is intuitive • The capacity to use on different platforms (mobile, desktop)	
**Doctors want**	
• A tool embedded within the patient management system • A tool that is fast • The capacity to turn on/turn off/ignore tool or parts of the tool • A simplified interactions checker (so prescriber doesn’t need to check each medication individually) • A tool targeted to reduce polypharmacy • Age and renal function integrated into any calculations • Children’s weight integrated into prescribing calculations (and printed on label) • Pregnancy or pregnancy risk factored into recommendations • Ethnicity (and earlier onset of disease) factored into risk weightings • A simple risk severity grading system (e.g. traffic light system) • Other risk assessment tools integrated within the one tool (e.g. CHA_2_DS_2_-VASc) • Patient access to empower shared decision-making • Streamlined monitoring for medications • An audit function to the tool • Alerts which are highly clinically relevant • Alternative medication suggestions (e.g. current first line agents based on updated prescribing or antimicrobial guidelines)	
**Patients want**	
• Access to the tool independent of their doctor • A tool that is culturally sensitive (and perhaps the potential to change language) • Something that is free to use and will help them understand their medications • “Just right” amount of information: not too much nor too little • Risk information presented simply (e.g. traffic light system)	

Patients were more positive than doctors about the concept of the tool. Patients envisaged the tool would provide them with trustworthy medical information that they could access both during their general practice consult and also later, independently of their doctor. They felt this would facilitate information sharing and decision-making with whānau. Patients equated medical knowledge with improved understanding and control of their health:

“I’m surprised that doesn’t already exist... I think what is really important is giving people the mana or the authority over their experience and their information and their decisions. I think everything else stems from that.” Patient 1 (Māori female)

“So in terms of that decision making, some sort of tool that would allow me to know what it was I was taking and why would be helpful… And I suppose this is a more difficult one, I suppose red flags in terms of there are things that you should be keeping an eye on with this medication.” Patient 2 (Māori male)

However, both doctors and patients were concerned there was potential for the tool to exacerbate harm. Too much information could put people off taking their medication or induce the nocebo effect:

“How in-depth do we need to go? There are so many side effects, and we can impose our expectations of what patients might experience.” Prescriber 6 (European male)

“It would be useful, but I think it could potentially scare someone off having a medication.” Patient 10 (Māori female)

“If there was some kind of unintentional bias in the way medicines were talked about and explained, you might end up, I don’t know, with one of two options sounding much more attractive than the other… so I think there could be unintended consequences of not being neutral about it.” Patient 1 (Māori female)

The form of the tool was debated by participants. Doctors mainly discussed access from their perspective, access directly integrated into their electronic health record system being heavily preferred over a separate add-on website. Most patients thought some kind of app or secure website, such as accessing information via their patient portal, would be most useful and provide independent patient access. (In NZ, patient portals currently allow patients variable access into their own general practice records, depending on what their practice has chosen. Access ranges from minimal—booking appointments online and checking blood results, to open notes—where patients can see all parts of their record). Older patients stated they would not be able to access information if it was a technology-based tool. This barrier was recognised by most participants:

“Maybe just the people who are not good with technology might struggle quite a lot.” Patient 8 (European female)

### Theme 3—“Just to be greeted with ‘Kia ora!’” [Collective Action]

Collective Action is underpinned by the concept of relational integration, which explores the effect of the intervention on human relationships, especially the effects on power and trust [45, 57]. Although relationships were not a particular focus of the interview questions, this theme was discussed in detail by all participants. They recognised that the action of establishing a relationship between patient and prescriber is required before any meaningful use of a prescribing tool or shared decision-making can take place.

Patients were asked what features would enhance the tool and its use for them. The use of Māori and Pacific languages was seen as important for enhancing engagement and understanding both within the tool and when communicating with the clinician. Whānau are important contributors to decision making; therefore, opportunities for including whānau need to be incorporated into decision making processes when using the tool:

“Just to be greeted with ‘Kia ora!’ Little things like that make a big difference, I think when engaging with a clinician.” Patient 4 (Māori male)

“Some people don’t understand English. But having that written in their own language and they read it and understand it… I know some seniors that depend on their children or grandchildren to tell them and describe how to take the medicine or when to take the medicine. So I reckon that is really important.” Patient 14 (Pasifika female)

“I think it’s just important to include whānau in decision making. That’s really important, which I think is not just cultural, it just should be done anyway. A collaborative approach.” Patient 4 (Māori male)

Patients wanted clinicians to use culturally safe practices and to acknowledge that people of different cultures may not feel comfortable attending general practice. The power imbalance between patients and clinicians was thought to be exacerbated by traditionally deferential attitudes towards clinicians, especially if they were of a different gender to the patient. These factors can obstruct discussions of medication risk and shared decision-making, particularly in the time-limited setting of a medical consultation:

“I think there’s lots of different cultural things about going to the doctor and Māori really feel a lot of whakamā [shyness/embarrassment] when talking about some stuff or depending on the doctor they get.” Patient 1 (Māori female)

“Sometimes in a way embarrassed to talk to the doctor. Our culture is a respect. Yeah. It’s a culture is a respect to talk to the doctor, especially the woman talk to the man, the doctor, man doctor.” Patient 13 (Pasifika female)

“When, as a traditional Pasifika person, you’re told, ‘This is what’s going to happen. You’re going to get this. And I know, because I’m the doctor, and I’m telling you that this medication will help whatever, your lumbago or hypertension or whatever it is.’ All you say as a traditional Pasifika person, ‘Yes, doctor. Yes, yes.’ As soon as you walk into that room, as soon as you walk through that door, the stethoscope or the persona of the person, or whatever they’re wearing, takes away your right of questioning, of understanding.” Patient 12 (Pasifika male)

Trust was acknowledged by both clinicians and patients as an important contributor to shared decision-making. While patient-centred care encourages patients to play an active role in their healthcare, the traditional approach of relying on the doctor’s opinion is still an important factor in decision making:

“They trust us as their doctors… I can spend a lot of time discussing the pros and cons and they end up saying, ‘Well what would you do, what do you advise?’” Prescriber 3 (European male)

“I tend to rely on my GP, I’ve got quite a high degree of trust for them to be able to manage that that risk for me.” Patient 2 (Māori male)

“We’ve got a really great relationship. So I feel that he has got my best interest in mind when he prescribes something to me.” Patient 10 (Māori female)

A tool that provided clinicians with tailored risk information and promoted communication of that information in a culturally safe and respectful way could enhance the doctor-patient relationship by facilitating shared decision-making. One patient thought the tool could potentially redress some of the power imbalance:

“It might be a model that would shift to having to patients having more power, I suppose than, rather than traditional is going to the doctor because you’ve got a sore throat and you come out with a prescription… I always think it’s good when people personally have more power over what they need for themselves.” Patient 5 (European female)

## Discussion

This research was conducted to determine what participants think about a proposed electronic prescribing, decision-support and communication tool. Doctors and patients prospectively evaluated a theoretical tool in order to refine the design. The main findings are broadly consistent with existing research.

In theme 1 (NPT = Coherence), patients and doctors understood the underlying premises of the proposed tool, particularly the tension between benefits and risks of prescribing. Few patient participants felt they had a good understanding of their medications, as has been found previously [58, 59]. Patients were able to clearly describe examples of both poor and excellent risk communication. An important finding is that patients preferred full disclosure of medication risks in a manner that they can understand. In contrast, doctors felt they give an adequate amount of information about medication and risk, based on their personal assessment of their patients, which may well reflect their own biases and exacerbate inequity [60]. These findings are consistent with previous research exploring patients’ and doctors’ attitudes towards information sharing [61–65]. As has been found elsewhere, doctors in our study typically found it difficult to communicate risk [66–68].

Participants actively evaluated the tool in theme 2 (NPT = Cognitive Participation), offering many suggestions as to how the tool could best suit their needs. Clinicians described existing ineffective or unworkable tools as models to avoid. They would accept a tool as proposed only if it was useful and was not perceived to cause additional work at the time of the initial clinical encounter (although if properly implemented the tool has potential to reduce their workload through the prevention of harms requiring further clinical review). Their statements echoed the vast body of literature outlining failed e-tools and alert fatigue [69–72]. It is critical software developers ensure the benefits of using any tool outweigh the clinical disruption associated with its use [53]. Also consistent with existing research, patients want reputable medicines information that they can access on their own terms and in their own time [58, 61, 73, 74]. Patients and clinicians were concerned there was a potential for the tool to generate negative health outcomes mainly as a result of the nocebo effect; extant literature demonstrates both that these concerns have been shared by others [61] and the validity of both nocebo and placebo effects [75, 76]. It is difficult to differentiate between a nocebo effect and patients accurately identifying adverse effects they have been warned about. Concerns were also raised about technology being a barrier for some patients, which is a known problem [77, 78].

In theme 3 (NPT = Collective Action), patients and doctors stressed the pre-eminence of establishing culturally safe relationships. Cultural safety is recognised as an independent requirement for achieving health equity [9, 79]. Participants emphasised the importance of communication, particularly the use of Māori and Pacific languages to facilitate understanding both in clinical settings and in the proposed tool. This is congruent with known strategies to improve cross-cultural communication, such as clinician training and enhanced use of interpreter services [49, 80]. Trust remains a bedrock of the doctor-patient relationship; without shared power, this approach does not promote shared decision-making [81]. Patients want to play an active part in decision-making about their health, while clinicians felt there was a wider range of patient responses—some patients have no interest in shared decision-making. The literature appears to support both perspectives [62, 82–84].

The fourth NPT concept of Reflexive Monitoring [52], which ascertains to the impact of a tool, was considered less relevant to this prospective evaluation of a potential tool. Patients and GPs were asked to imagine the potential system-wide effects of the tool, as well as the intended and unintended consequences that might arise from its use. Participants found these questions difficult to answer, therefore there was little relevant data pertaining to this concept.

### Strengths and limitations

This study successfully determined what participants think about a proposed risk assessment and communication tool, and interprets this through the lens of NPT. This prospective assessment of the tool will be used to refine the proposed tool to ensure it meets the requirements of prescriber and patient end-users. This study lays the groundwork for future analysis of the tool as it progresses through development and testing phases. Future analyses will be able to focus on more practical elements of NPT review. Ultimately, it is hoped that the use of the proposed tool will support patient understanding of their risk of harm from medication, facilitate shared decision-making and improve the quality of informed consent, while not increasing health inequity. This research was designed to inform the development of a risk assessment and communication tool in NZ; therefore, the findings may not be generalisable beyond this scope.

Participants were all volunteers and may not represent typical patients and doctors. Due to recruitment via Facebook, it is not known how many people chose not to participate. Patient participants were highly engaged in their healthcare; all patient participants wanted more information about their medications and participating in shared decision-making. Conversely, prescriber participants reported a far wider range of patient interest in active participation in their healthcare. Similarly, prescriber participants were those who were interested in quality improvement and healthcare technology and might not represent typical clinicians.

Satisfactory ethnic diversity was obtained for patients; however, each ethnicity group is not comparable in terms of education and background; NZ European patients were mainly unemployed or students, Māori patients in this study were typically highly educated, while the Pasifika patients were predominantly immigrants to NZ who spoke English as a second language. Patients therefore were not fully representative of their ethnic group, and this may limit the extent to which their views and experiences reflect the full range of views and experiences within their ethnic group. For example, traditional deferential attitudes towards doctors were discussed by Pasifika participants, but these attitudes are not universal amongst Pasifika peoples, particularly younger people and those born in NZ. Ethnic diversity amongst doctors was not intentionally sought and was consequently limited. Of the nine doctors, only two were Māori, and there were no Pasifika doctors.

NPT was used as a framework for developing the questions and as a sensitising device developing the codes and themes (Fig. 1). NPT was useful in this context; however, our research findings suggest the emphasis of this framework could be rearranged slightly to augment the construct of Coherence. Theme 1 strongly suggests personal or whānau experience is a critical factor in understanding medication harms for patients and to a lesser degree for doctors. The lived experiences that participants bring to the sense-making work of establishing coherence is not explicitly recognised within the construct of Coherence as it is currently defined.

Participants were united in highlighting the primacy of relationships in the context of healthcare provision and use of any prescribing and communication tool. Relational Integration is included within NPT construct of Collective Action, but is only ranked second of the four elements that make up this construct. In earlier iterations of NPT, Relational Integration and the other three elements now contributing to the construct of Collective Action formed the entire Normalisation Process model [85]. It may be that when implementation of a patient-facing intervention is planned using NPT, the area of relational integration requires more emphasis.

### Implications

Given Aotearoa New Zealand’s current high levels of inequity based on ethnicity and socioeconomic status, it is vital to consider and pre-emptively address the potential of any new intervention to worsen inequity [30, 86]. In general terms, “upstream” actions that focus on equity from a health policy or systems perspective, such as improving access by reducing co-payments for healthcare, have a far greater impact on equity than “downstream” interventions, such as education of individuals, or the use of a tool like that proposed [86, 87]. However, co-designing interventions tailored for the needs of different groups can reduce barriers to receiving healthcare and has the potential to reduce inequities arising from use of technological interventions [78, 88]. It is likely that a multidimensional approach is required to reduce health inequities, founded on culturally safe and trustworthy relationships [89]. Targeted strategies to increase technology use can go some way to bridge the digital divide, such as public provision of computers and internet access, while family and clinician support can encourage older patients to use technology [73, 78].

## Conclusion

Patients and doctors provided different perspectives when evaluating a proposed risk assessment and communication tool. Patient participants were keen to take an active part in their health and participate in shared decision making about their healthcare, whereas doctors described a wider range of interest in patient participation. NPT was a useful theoretical framework to conduct this evaluation and identify both requirements for the tool and features to avoid. This co-design research identified ideas for the proposed tool which had not been previously considered, such as providing patients with access to information about their medicines independently of their doctor. Overall patient and doctor participants supported the development of the proposed risk assessment and communication tool, but recognised successful use of the tool requires culturally safe and trustworthy doctor-patient relationships. The use of Māori and Pacific languages in the proposed tool may enhance engagement and understanding.

## Supplementary Information

**Additional file 1 **: **Appendix 1**. Additional information as per the consolidated criteria for reporting qualitative research (COREQ) checklist.

**Additional file 2 **: **Appendix 2.** Interview Topic Guide.

## Data Availability

Supporting data (interview transcripts) are not available for review as they contain information which may identify participants. A summary of anonymised supporting data are available from the corresponding author on reasonable request.

## Abbreviations

GP: General practitioner
HRC: Health Research Council of New Zealand
NPT: Normalisation Process Theory
