# Supplementary material for: The views of New Zealand general practitioners and patients on a proposed risk assessment and communication tool: a qualitative study using Normalisation Process Theory
Source: Implement Sci Commun. 2021 Feb 10;2:16. doi: 10.1186/s43058-021-00120-1 (PMC7877107; doi:10.1186/s43058-021-00120-1)
Supplement: Supplementary file 1 — Additional file 1 : Appendix 1. Additional information as per the consolidated criteria for reporting qualitative research (COREQ) checklist. [file 43058_2021_120_MOESM1_ESM.docx]

| **Appendix 1. Additional information as per the consolidated criteria for reporting qualitative research (COREQ) checklist** | |
| --- | --- |
| **Domain 1: Research Team and reflexivity** | |
| **Personal Characteristics**   1. Interviewer/facilitator; 2. Credentials; 3. Occupation; 4. Gender; Experience and Training | SL conducted all the interviews. SL is a general practitioner with 12 years of clinical experience and 5 years of health research experience. This work was completed as part of her PhD, supervised by TS and AS. SC is a research advisor on this project.  TS is an experienced researcher and general practitioner who has led and collaborated on externally funded (≈ NZ$7.8 million) health service delivery implementation research (UK & NZ) using both quantitative and qualitative methods. His research expertise in general practice and in quality improvement research is fundamental to this project.  AS is an experienced investigator in the field of clinical pharmacy, focussing on quality use of medicines and Pharmacoepidemiology. She has experience developing and implementing trials of electronic decision support tools.  SC is an experienced Māori health researcher and general practitioner. She helped plan the data collection, and provided cultural insights into the data analysis.  All researchers were employed by the University of Otago. SL is a Clinical Research Training Fellow, TS is a Professor, AS is a Senior Research Fellow, and SC is an Associate Professor. |
| **Relationship with participants**  6. Relationships established; 7. Participant knowledge of the interviewer; 8. Interviewer characteristics | Collegial relationships were well established with the local Dunedin prescribers prior to the commencement of the study, who were personally invited to participate by SL. Invitation was made either face-to-face or via email. Several of the participants recruited via Facebook page “GPs for GPs” were also known to SL.  Patients recruited for the study were unknown to SL. They were recruited via Facebook and Facebook Messenger, email or phone was used to arrange the interviews.    SL was identified to all participants as a GP who was conducting research for her PhD research. SL has had extensive communication skills training through her undergraduate, postgraduate and vocational training. She has 18 years’ experience working as a clinician, 12 years of those working as a GP. |
| **Doman 2: study design** | |
| **Theoretical framework**  9. Methodological orientation and theory; | The methodology of this paper is outlined in detail within the paper. It draws strongly on Implementation Science theory, particularly Normalisation Process Theory (NPT). |
| **Participant selection**  10. Sampling; 11. Method of approach; 12; Sample Size; 13. Non-Participation | A purposive sampling approach was taken for recruitment of both prescribers and patients. Initially Dunedin GPs were invited to participate in the study. This resulted in 5 face-to-face interviews with Dunedin-based prescribers. Subsequent prescribers were recruited on a closed Facebook page for New Zealand GPs, “GPs for GPs”. Five people, four men and one woman expressed an interest in participating in the study, with the four men all participating in either phone or Zoom interviews.  Patients of different ethnicities were sought for this project in an attempt to increase the diversity of the views. A Māori-owned company in Dunedin was approached for research participants, resulting in three interviews with Māori patients. Participants were then sought via a post on the “Dunedin News” Facebook page. This was an extremely effective method of recruitment, and a further two Māori patients, five NZ European patients, and one Pasifika patient were recruited. Pacific Trust Otago were then approached for recruitment. The Trust holds a weekly meeting for their elders, which includes exercise (provided by a physiotherapy student), food, a sing-along, and an occasional educational talk. SL gave an educational talk on medication safety at this meeting and recruited another four Pasifika patients. SL had no relationship with any patients prior to the study interview, apart from the Pacific Trust group as described.  All participants received information about the study and signed a consent form approved by the University of Otago Human Ethics committee (19/020) prior to their interview.  The study team anticipated that we would not need any more than 15 patient or GP interviews to reach data saturation in each group. SC suggested the ethnicity split 5 European/5 Māori/5 Pasifika as that would help us gain an understanding of the perspectives of different ethnicities.  Due to recruitment via Facebook, it is not known how many people chose not to participate. |
| **Setting**  14. Setting of data collection; 15. Presence of non-participants; 16. Description of sample | Dunedin-based prescriber interviews occurred face-to-face, in either the prescriber’s office, at the University of Otago, or in a café.  All patients were Dunedin-based to allow face-to-face interviews. These took place in the patients’ home, their workplace or at the University of Otago. Non-participants were not present during the interviews.  Participants self-identified their personal characteristics, including age and ethnicity |
| **Data collection**  17. Interview guide; 18. Repeat interviews; 19. Audio/visual recording; 20. Field notes; 21. Duration; 22. Data saturation; 23. Transcripts returned | SL developed the interview guide following the NPT structure. This was reviewed by TS and AS, and refined through practice interviews. No repeat interviews occurred, but two prescriber participants sent additional material to SL via email following their interview. All but one of the interviews were recorded, due to a failure of the recording equipment on one occasion. Extensive field notes were taken during that interview and were written up immediately afterwards. Each interview lasted from 30-90 minutes. Interviews were either transcribed by SL or sent to rev.com for transcription, and all transcripts were closely reviewed and revised by SL to ensure they were as accurate as possible. Participants did not have the opportunity to review transcripts.  Although the sample size was planned as executed, data saturation was observed within the last few interviews in each group, with no new ideas being discussed by participants. |
| **Domain 3. Analysis and findings** | |
| **Data analysis**  24. Number of data coders; 25. Description of the coding tree; Derivation of themes; 27. Software; 28. Participant checking | SL developed the coding structure and coded all data using NVIVO qualitative data management software(1). Coding was reviewed in depth by TS. Codes were clustered into themes derived from the NPT framework and focussed on the development of the proposed tool.  Participants were not involved at the data analysis stage. |
| **Reporting**  29. Quotations presented; 30. Data and findings consistent; 31. Clarity of major themes; 32. Clarity of minor themes | Quotations were extracted from the transcripts to illustrate the major and minor themes. These were checked during analysis to ensure they were not taken out of context of the interview, and consistently and accurately represented the participants’ views. |

1. NVIVO qualitative data analysis software. 11 ed: QSR International Pty Ltd.; 2015.
