# Supplementary material for: The views of New Zealand general practitioners and patients on a proposed risk assessment and communication tool: a qualitative study using Normalisation Process Theory
Source: Implement Sci Commun. 2021 Feb 10;2:16. doi: 10.1186/s43058-021-00120-1 (PMC7877107; doi:10.1186/s43058-021-00120-1)
Supplement: Supplementary file 2 — Additional file 2 : Appendix 2. Interview Topic Guide. [file 43058_2021_120_MOESM2_ESM.docx]

# Interview Topic Guide

## Interview Topic Guide for Prescribers

MedKōrero is a tool to assess and communicate medication risk, to promote shared decision making and potentially reduce the risk of patient harm.

| **Before we begin, do you have any further questions** (about the consent process/study)? |
| --- |
| **Could you tell me a bit about yourself and your practice setting?**  How long have you been at this practice?  How long have you worked as a GP (and in NZ if applicable)?  Have you completed any extra qualifications on top of your medical degree?  What is your list size? What is your role in the practice (partner/associate/locum)?  What PMS does your practice use? |
| **What prompts you to consider assessing a patient’s risk from their medication?**  How do you currently assess patients’ risk of medication harm?  What tools do you use?  Do you have support in this area from a pharmacist/secondary care? |
| **How confident do you feel explaining medication risk to patients?**  What encourages/discourages you to discuss risk with patients?  How open do you feel your patients are to discussing medication risk?  When would you focus on medication risks and when would you focus on the benefits? Why?  Do you use any resources to help explain concepts of risk to patients? What are they?  Risk scenarios:  *High risk patient*: elderly female, eGFR 20 mL/min, 10 long term medications, presents with increasing SOB likely due to deterioration of known congestive heart failure.  *High risk medication*: Methotrexate/Insulin/Warfarin  *High risk condition*: Psychosis/rheumatoid arthritis/cancer |
| **What do you understand by the term shared decision making?**  How do you feel about shared decision making in your clinical practice?  How do you promote shared decision making?  How open do you feel your patients are to shared decision making?  Have you ever had any training in promoting shared decision making with patients? |
| **Can you describe a case (anonymously) where a patient has experienced problems from their medication that were potentially preventable?**  What stands out for you in this case that was different from other patients?  What were the factors contributing to medication harm?  What were the factors contributing to patient safety?  You mentioned XXX as working well, do you think this could be supported in other cases?  You mentioned XXX as working poorly, how do you think this could be changed? |
| **Do you think the proposed MedKōrero tool, to assess risk and communicate that risk to patients, will promote shared decision making?**  What kind of impact would a tool like this have in your clinical setting?  Would it be helpful to your day-to-day work?  What would promote its use?  What would be a barrier to its use? |
| **What would be the top priorities for a patient safety tool like MedKōrero?**  High risk patients?  High risk medications?  High risk conditions? |
| **Can you think of potential system-wide effects of using this tool?**  What would be the intended and unintended consequences |
| **If you could make a recommendation/suggestion to improve patient safety in primary care in relation to medication use, what would it be?**  Explore patient factors in relation to these recommendations for larger implications |
| **Can you think of anything else I should ask?**  Is there anything else I should ask other prescribers? |

## Interview Topic Guide for Patients

MedKōrero is a tool to assess and communicate medication risk, to promote shared decision making and potentially reduce the risk of patient harm.

| **Before we begin, do you have any further questions** (about the consent process/study)? |
| --- |
| **Could you tell me a bit about yourself and your experiences with medicine?**  How old are you? Ethnicity?  Do you take medicine every day?  When was the last time you were prescribed medicine?  Do you help someone else regularly with their medication? |
| **What does harm from medicine mean to you? What does risk from medicine harm mean?**  Can you give me an example?  Synonyms of risk: danger, peril, possibility, hazard, menace, threat |
| **When do you think it’s important to know about your risk from medication?**  High-risk vs low-risk medications/conditions?  Treatment vs prevention?  Personal perception of risk (high risk patient)? |
| **Do you think it’s important to discuss medication risk with your GP?**  What encourages/discourages you to discuss risk with your GP?  How open do you feel your GP is to discussing medication risk?  When would you focus on medication risks and when would you focus on the benefits? Why?  Have you ever been given information about medication risk? What was it? Was it helpful?  Risk scenarios:  *High risk patient*: elderly relative who is frail and on 10 long term medications.  *High risk medication*: Methotrexate/Insulin/Warfarin  *High risk condition*: Psychosis/rheumatoid arthritis/cancer |
| **What do you understand by the term shared decision making?**  Can you recall a time when you felt like you and your doctor made treatment decisions about your health care together?  How do you feel about shared decision making about your health care?  How open do you feel your doctor is to shared decision making? |
| **Can you describe a time when you or someone close to you experienced problems from their medication?**  What were the factors contributing to the medication harm?  What were the factors contributing to patient safety?  You mentioned XXX as working well, do you think this could be supported for other people?  You mentioned XXX as working poorly, how do you think this could be improved? |
| **Do you think the proposed MedKōrero tool, to assess risk and improve communication about that risk, will help you/your whanau make decisions about treatment?**  Do you think it would promote shared decision making?  What kind of impact would a tool like this have for you/your whanau when you are deciding on a treatment option?  What would promote its use?  What would be a barrier to its use? |
| **What do you think should be the top priorities for a patient safety tool like MedKōrero?**  High risk patients?  High risk medications?  High risk conditions? |
| **Can you think of potential system-wide effects of using this tool?**  What would be the intended and unintended consequences |
| **If you could make a recommendation/suggestion to improve patient safety in primary care in relation to medication use, what would it be?**  Explore patient factors in relation to these recommendations for larger implications |
| **Can you think of anything else I should ask you?**  Is there anything else I should ask other people? |
